# Ion selectivity of graphene nanopores

**DOI:** 10.1038/ncomms11408

**Published:** 2016-04-22

**Authors:** Ryan C. Rollings, Aaron T. Kuan, Jene A. Golovchenko

**Affiliations:** 1 Department of Physics, Harvard University, Cambridge, Massachusetts 02138, USA; 2 School of Engineering and Applied Sciences, Harvard University, Cambridge, Massachusetts 02138, USA

## Abstract

As population growth continues to outpace development of water infrastructure in many countries, desalination (the removal of salts from seawater) at high energy efficiency will likely become a vital source of fresh water. Due to its atomic thinness combined with its mechanical strength, porous graphene may be particularly well-suited for electrodialysis desalination, in which ions are removed under an electric field via ion-selective pores. Here, we show that single graphene nanopores preferentially permit the passage of K^+^ cations over Cl^−^ anions with selectivity ratios of over 100 and conduct monovalent cations up to 5 times more rapidly than divalent cations. Surprisingly, the observed K^+^/Cl^−^ selectivity persists in pores even as large as about 20 nm in diameter, suggesting that high throughput, highly selective graphene electrodialysis membranes can be fabricated without the need for subnanometer control over pore size.

## Abstract (web summary)

Sub-nanometer graphene nanopores are usually required to create graphene-based reverse osmosis membranes. Here, Rollings *et al.* show that membranes with larger pores are highly ion selective and a hundred times more permeable to potassium ions than to chloride ions, making them useful for electrodialysis.

## Graphical abstract

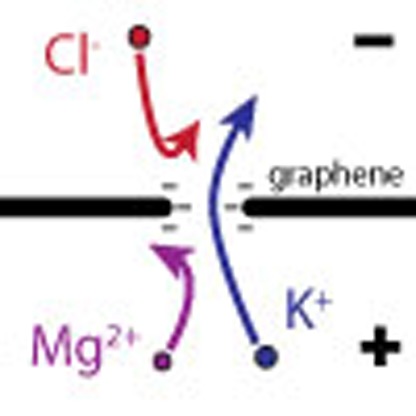

Atomically thin graphene membranes have generated considerable interest for use as filtration membranes because their atomic thickness presents minimal resistance to fluid or ion flow while retaining high structural integrity. Recent investigations have suggested that porous graphene membranes can attain orders of magnitude higher flow rates than commercial reverse osmosis (RO) membranes, while still providing excellent salt rejection^1^,^2^,^3^,^4^,^5^,^6^,^7^. Unfortunately, RO salt rejection depends on a very tight distribution of subnanometer pores. A few large pores in a membrane can contribute large unselective water fluxes, impairing salt rejection. Thus, viable RO membranes depend on the complete elimination of pores larger than a nanometre or so, which remains a difficult fabrication challenge^6^,^8^,^9^. However, recent theoretical predictions suggest that graphene nanopores that are too large for RO may still be suitable for electrodialysis if they are electrostatically charged, allowing them to separate anions from cations^10^,^11^,^12^. Yet, experimental investigations of ion selectivity of graphene nanopores have been limited to subnanometer pores^7^,^13^,^14^.

Here, we examine ion selectivity (cations versus anions, as well as among different cations) of single graphene nanopores with an emphasis on the relationship between ion selectivity and pore size. These experiments not only allow us to evaluate porous graphene membranes as electrodialysis membranes, but also shed light on the chemical structure of graphene pore edges and ion-specific interactions with graphene membranes.

## Results

### Nanopore fabrication

**Figure 1:**
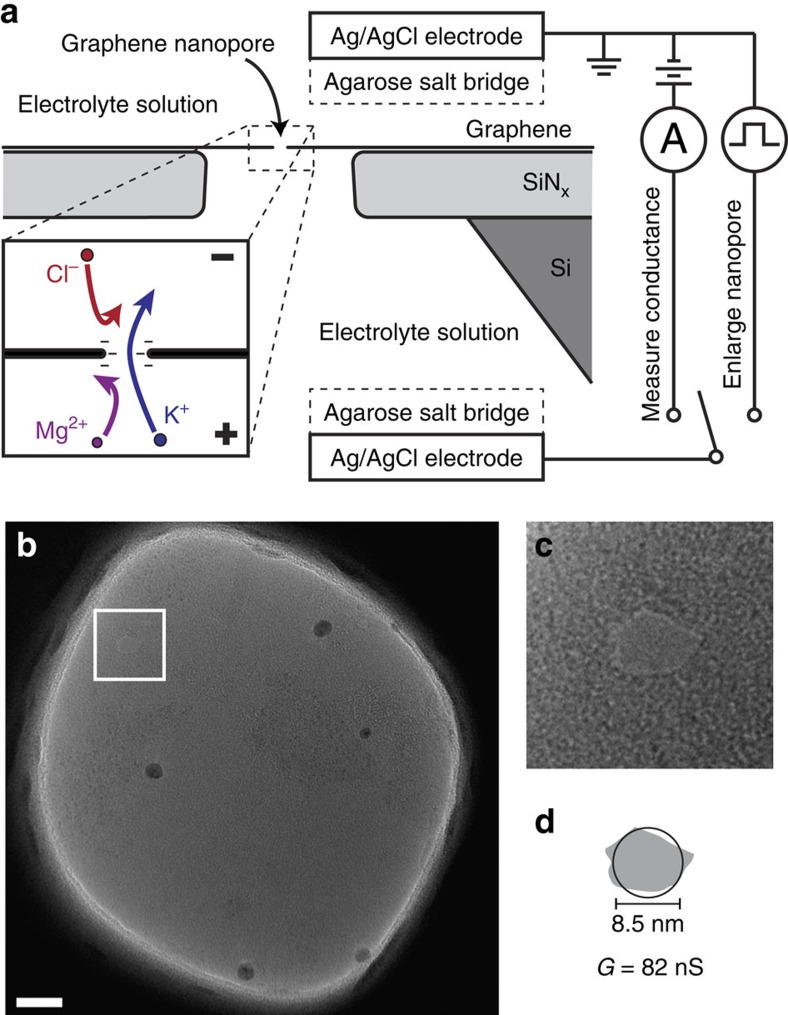
Experimental setup. (**a**) Cross-sectional diagram of a suspended graphene nanopore sample immersed in electrolyte solution. Ag/AgCl electrodes that contact the solution via agarose salt bridges are used to measure ionic current through the nanopore or enlarge the nanopore using electrical pulses. Inset shows an illustration of anion and divalent cation rejection in a negatively charged nanopore. (**b**) TEM image of a suspended graphene membrane after a pore has been created via electrical pulse fabrication. Scale bar, 20 nm. White box indicates the location of the nanopore. (**c**) Close-up of area containing a nanopore. The sides of the image are 30 nm in length. (**d**) Comparison of the size of the pore with estimation of the pore size calculated from the pore conductance via equation 1. The grey outline is traced from the TEM image and the black circle is calculated from the conductance *G*.

Graphene nanopores were fabricated using a recently reported electrical pulse method that enables rapid fabrication of very small single nanopores, as well as controllable, *in situ* enlargement of the nanopore^15^. This method allows measurements to be performed for multiple pore sizes with a single sample, which would be extremely difficult and time consuming using electron-beam drilling fabrication methods^16^,^17^,^18^,^19^,^20^. To create a pore, freestanding graphene membranes were placed in a flow-cell between two fluid reservoirs filled with 1 M KCl as schematically depicted in Fig. 1a. Ultra-short, high voltage pulses were applied across the membrane to nucleate and enlarge single nanopores. A transmission electron microscope (TEM) image of a graphene membrane after electrical pulse fabrication is shown in Fig. 1b. The outline of the pore can be clearly seen in a close-up of the image shown in Fig. 1c. Although TEM imaging can be used to measure pore sizes as was done here, preparing and imaging the samples after solution-based experiments is labour-intensive and low-yield (see the Methods section). Therefore, for the bulk of our experiments, we estimated the pore size based on the measured conductance of the nanopore in 1 M KCl solution using an analytical model of pore conductance^15^,^16^,^21^,^22^, given by


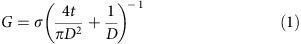


where, *G* is the pore conductance, *σ* is the solution conductivity (105 mS cm^−1^), *t* is the effective thickness of the graphene membrane (0.6 nm, see ref. 16), and *D* is the pore diameter. Figure 1d shows the outline of the pore obtained via TEM imaging (grey) compared with the estimated size of the pore, based on the conductance of the pore in 1 M KCl at pH 2 (black circle). The close agreement suggests that equation 1 does an adequate job of estimating the pore size, precluding the need to image every sample with TEM. While the exact mechanism that produces electrically pulsed nanopores is not yet fully understood, it likely involves the oxidation of carbon at the pore edge^15^, which results in carboxyl or other protonatable edge groups^23^ that bestow a negative charge on the edge of the pore at neutral and higher pH (Fig. 1a, inset). Previous research on comparatively thick, insulating solid-state nanopores has demonstrated that negative charge at the periphery of the pore repels anions and attracts cations, which conduct the bulk of the ionic current^24^,^25^,^26^,^27^. However, such electrostatically controlled ion selectivity was thought to be negligible for pores in which the diameter is significantly larger than the membrane thickness^28^. Indeed, recent measurements have shown that sub-2 nm intrinsic defects in chemical vapour deposition (CVD) graphene membranes can distinguish between mono and divalent cations^14^, but comparable measurements for larger pores have not been performed.

### Cation/anion selectivity

**Figure 2:**
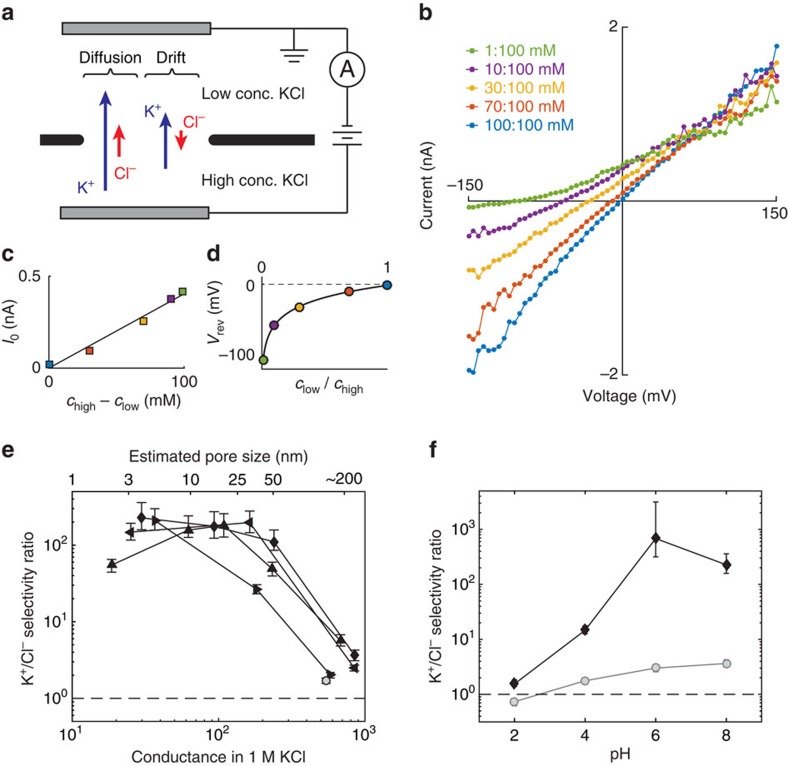
K^+^/Cl^−^ selectivity. (**a**) Schematic of experimental setup: a concentration gradient and electric potential are simultaneously imposed across the nanopore and the net ionic current is measured. (**b**) Measured *I-V* curves for several concentration ratios. Each coloured curve indicates a different concentration ratio as indicated in the legend. (**c**) Zero-bias current indicates that K^+^ ions pass more easily than Cl^−^. Markers are coloured the same as in **b**. Solid line is a linear fit. (**d**) Reversal voltage as a function of concentration ratio, along with fit to the GHK voltage equation (solid line, equation 1), which is used to calculate selectivity. Markers are coloured the same as in **b**. (**e**) K^+^/Cl^−^ selectivity ratio as a function of pore size for several nanopores; different markers indicate different samples. The conductance in 1 M KCl (lower *x*-axis) is used to calculate the pore diameter (upper *x*-axis). Error bars indicate the 5th and 95th percentile estimates. The open circle indicates a control aperture with no suspended graphene. Dotted line indicates no selectivity. (**f**) K^+^/Cl^−^ selectivity ratio as a function of pH for a 3 nm pore (black diamonds), showing that selectivity increases with pH. In contrast, a sample with most of the graphene removed (grey circles) shows little selectivity at any pH. Error bars indicate the 5th and 95th percentile estimates. Dotted line indicates no selectivity.

To measure cation/anion selectivity, current-voltage (*I-V*) curves were performed with a variety of KCl concentration gradients across the pore (Fig. 2a). The voltage bias was applied via Ag/AgCl electrodes contacting the fluidic cell via agarose salt bridges, which are used to eliminate the potential generated from redox reactions on electrodes in different salt concentrations. K^+^ and Cl^−^ ions were selected as a representative cation/anion pair because they have very similar bulk mobilities (see Supplementary Table 1), and therefore exhibit negligible liquid junction potentials, about 1 mV for our measurements (as calculated by the Henderson equation^29^). Therefore, within experimental error (5 mV), the measured voltages were equal to the voltage drop across the graphene membrane. An example set of *I-V* curves for a 3 nm pore is shown in Fig. 2b. When there is no applied voltage (*V*=0) both K^+^ and Cl^−^ ions diffuse from high to low concentration, and a net current (short-circuit current, *I*_0_) is produced only if one ion diffuses at a higher rate than the other through the pore (Fig. 2c). The direction of this short-circuit current is consistent with the net flow of positive charges from high to low concentration, immediately indicating that the pore is cation selective. While the short-circuit current can identify selectivity, it is not a direct quantitative measure of selectivity because it also depends strongly on the conductance of the nanopore. A better choice is the reversal potential *V*_rev_, the applied potential at which the net current is zero (Fig. 2d), which in the well-known Goldman–Hodgkin–Katz (GHK) model does not explicitly depend on pore size^30^,^31^. By assuming that each ion species contributes a current given by the Nernst–Planck equation, which is parametrized by an effective diffusion constant 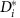 that is different for each ion species i, the GHK model presents a quantitative measure of selectivity that is useful for comparing selectivity among different pores^24^,^26^,^30^. In this context, the selectivity ratio *S*_GHK_ is defined as 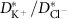 , which is equal to the ratio of drift currents from each ion when there is no concentration gradient. The reversal potential is related to the selectivity of the pore via the GHK voltage equation


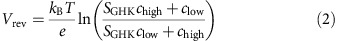


where *S*_GHK_ is the selectivity ratio, *c*_high_ and *c*_low_ are the solution concentrations in the fluid reservoirs, *e* is the electron charge, *k*_B_ is the Boltzmann constant and *T* is the solution temperature.

The K^+^/Cl^−^ selectivity ratio *S*_GHK_ was calculated for each pore by fitting the reversal potentials to the GHK voltage equation (equation 2, Fig. 2d). Figure 2e shows the selectivity ratio *S*_GHK_ plotted as a function of pore size for four samples at pH 8 (see the Methods section for error estimation). The lower horizontal axis is the measured conductance in 1 M KCl, while the upper axis shows estimated pore diameter based on equation 1. The K^+^/Cl^−^ selectivity ratios at pH 8 were generally above 100, values comparable to the biological ion channels and polymer membranes many microns thick^32^. Surprisingly, the selectivity ratios were not significantly reduced until the pores were larger than about 20 nm. These high selectivities contrast with previous measurements by O'Hern *et al.*^13^ on graphene membranes with many subnanometer pores, which reached a maximum selectivity ratio of *S*_GHK_=1.3. It is possible that the high K^+^/Cl^−^ selectivities measured here are unique to electrically pulsed pores, or that a small number of large pores or tears in the centimeter-scale membranes of O'Hern *et al.*^13^ drastically reduced selectivity by introducing parallel paths of non-selective ion flow. Indeed, pores larger than 100 nm (where the graphene pore is almost as large as the supporting silicon nitride aperture) showed minimal selectivity (*S*_GHK_<5, Fig. 2e). A control aperture with no graphene yielded *S*_GHK_≅2 (Fig. 2e, open circle), indicating that the aperture itself contributes non-zero but comparatively minimal selectivity. *S*_GHK_ was also measured at different solution pHs to examine whether or not K^+^/Cl^−^ selectivity is influenced by protonation/deprotonation of chemical groups (Fig. 2f). The measured selectivity for a 3 nm pore drops significantly between pH 6 and 4, and is negligible by pH 2. This pH dependence has a similar progression to deprotonation of edge groups expected at graphene edges (such as carboxyls)^33^, suggesting that the deprotonation of chemical edge groups is necessary for cation/anion selectivity.

While the presence of negatively charged groups on the pore edge can explain strong K^+^/Cl^−^ selectivity in subnanometer sized pores, it cannot account for larger pores (radius>Debye length≅1 nm in 100 mM KCl), where the edge charge is screened out by mobile counterions^25^,^28^. Moreover, in ultra-thin nanopores larger than a few nanometres in diameter, the pore conduction is mostly determined by access resistance extending out into the fluid^16^,^21^,^22^, which is largely unaffected by the charge at the edge of the pore. Therefore, a different mechanism is needed to explain the high selectivities for large nanopores. In the Discussion section, we will present a new model of pore selectivity that is consistent with our K^+^/Cl^−^ selectivity measurements.

### Inter-cation selectivity

**Figure 3:**
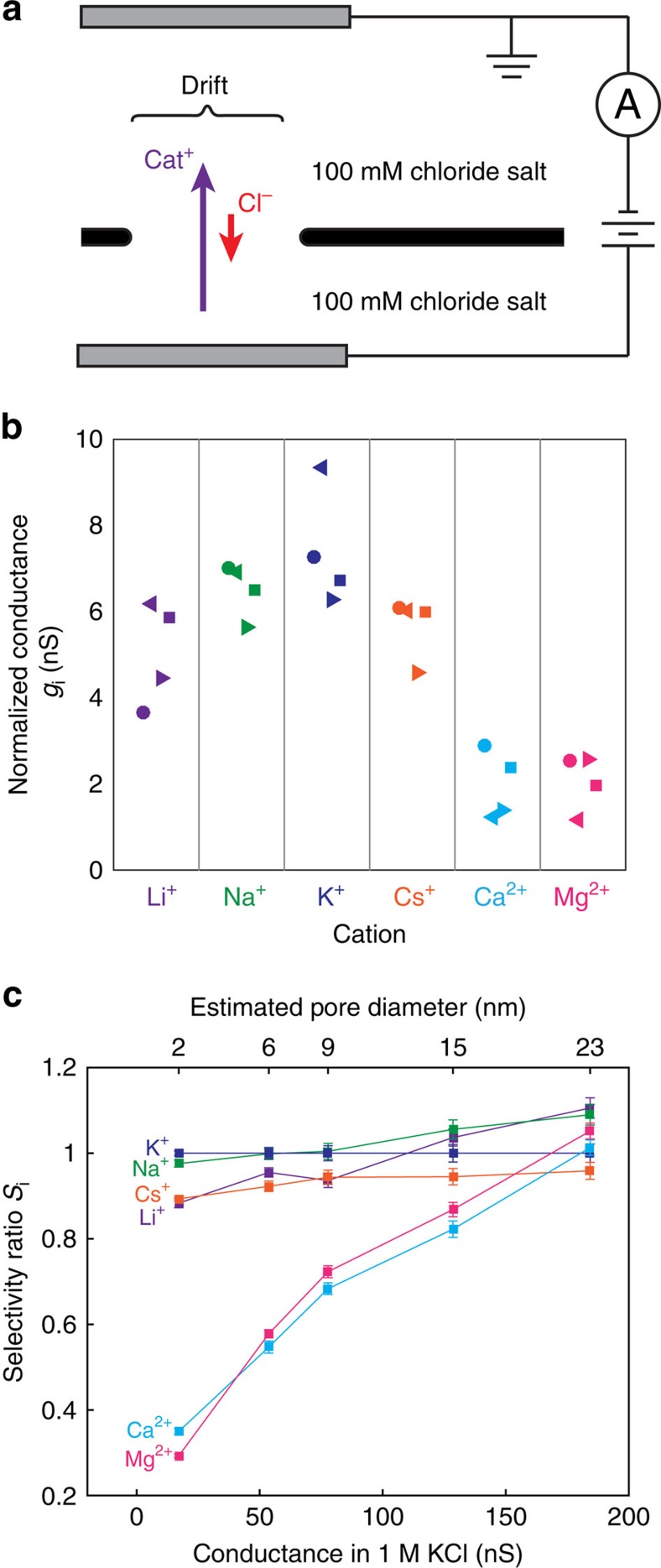
Inter-cation selectivity. (**a**) Schematic of experimental setup: pore conductance is measured in a variety of 100 mM cation-chloride solutions. (**b**) Normalized conductance *g*_i_ (see equation 3 for definition) for four different nanopores 2–4 nm in diameter; different shaped markers indicate different samples. Data is sorted by cation. Monovalent cations pass more easily than divalent cations. (**c**) Inter-cation selectivity ratio *S*_i_ (see equation 4 for definition) of a graphene nanopore as a function of pore size. Error bars indicate the standard deviation. The conductance in 1 M KCl (lower *x*-axis) is used to estimate pore size using equation 1. Inter-cation selectivity decreases as pore size increases and is no longer significant above 20 nm.

To measure selectivity among different cations, including divalent cations, we directly compared pore conductance in different cation-chloride solutions without a concentration gradient (Fig. 3a). This direct comparison can be made because the current due to Cl^−^ anions at pH 8 is negligible for small pores (as shown in the previous section). It is important to note, however, that different cations have significantly different electrophoretic mobilities, which result in differences in conductivity in bulk solution (see Supplementary Table 1). To account for these differences we introduce a normalized conductance *g*_i_ for each cation


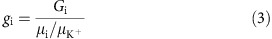


where, *G*_i_ is the measured nanopore conductance in cation-chloride solution, *μ*_i_ is the bulk electrophoretic mobility of the cation and *μ*_K^+^_ is the mobility of K^+^ ions. Any observed differences in normalized conductance indicate inter-cation selectivity of the pore. Figure 3b shows normalized conductances for a variety of mono and divalent cations for several small nanopores 2–4 nm in diameter. It is immediately evident that divalent cations show much lower normalized conductance than monovalent cations, which agrees with the recent results on sub-2 nm defects in CVD graphene membranes^14^. Even among monovalent cations, the normalized conductances appear to follow the general trend K^+^>Na^+^>Cs^+^>Li^+^. These results indicate that graphene nanopores can distinguish strongly between mono and divalent cations, and weakly among monovalent cations.

To examine how inter-cation selectivity depends on pore size, we measured normalized cation conductances for a pore during the sequential stages of electrical pulse enlargement (Fig. 3c, see the Methods section for error estimation). To characterize the relative selectivities for different cations, we define the inter-cation selectivity ratio (relative to K^+^) as


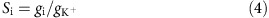


This definition of selectivity gives us *S*_i_=1 for a nanopore that does not distinguish between a cation i and K^+^. As the pore is enlarged, the same ordering K^+^>Na^+^>Cs^+^>Li^+^>>Ca^2+^>Mg^2+^ is preserved, but the selectivity of all cations is reduced as the pore size increases. For pores larger than 20 nm, no significant inter-cation selectivity remains. The deviations of *g*_i_ from 1 for such large pores are likely due to chlorine flux, which begin to contribute to the current for pores larger than 20 nm in diameter.

## Discussion

The persistence of strong K^+^/Cl^−^ selectivities in pores as large as 20 nm is surprising, and suggests that previously uncharacterized mechanisms may be responsible. We propose that the graphene surface (not just the pore edge) carries a pH-dependent surface charge due to deprotonatable oxygen-containing chemical groups on the graphene surface (oxidized graphene), or attached to the hydrocarbon contaminants on the graphene surface^34^,^35^. This surface charge, which would be negative at neutral pH values and neutralized in acidic solutions, would attract a screening cloud of positive counterions while also repelling anions in solution. Because these mobile cations near the surface would be concentrated relative to anions, they would contribute a large cation-selective ion current, causing the total ionic current to be cation-selective.

**Figure 4:**
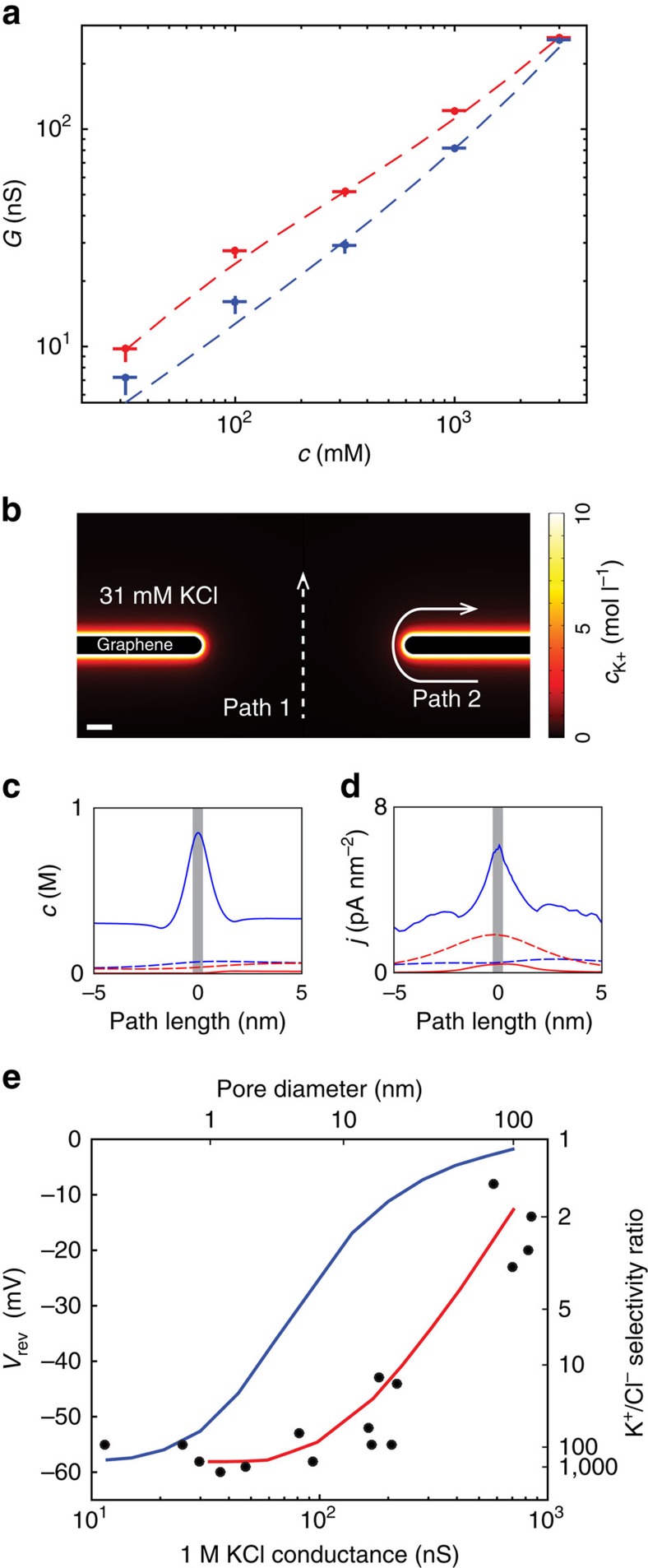
Surface charge model of ion selectivity. (**a**) Conductance measurements as a function of KCl concentration for an 8.5 nm pore (the same pore shown in Fig. 1b–d). Blue and red markers show experimental data at pH 2 and pH 8, respectively. Vertical and horizontal error bars indicate standard deviation. Dotted lines show numerical predictions from a numerical PNP model (see Supplementary Fig. 1 for details) with surface charge density of 0 C m^−2^ (blue) and −0.6 C m^−2^ (red). (**b**) Diagram of the numerical PNP system with negative surface charge (*σ*=−0.6 C m^−2^). Scale bar, 1 nm. The K^+^ concentration is plotted in a 31 mM KCl environment with 100 mV applied across the pore. Two illustrative current paths are indicated, path 1 (dotted line) through the centre of the pore and path 2 (solid line) along the membrane surface. (**c**) K^+^ (blue) and Cl^−^ (red) concentrations plotted along the two illustrative paths shown in **b**. The grey bar indicates the thickness of the nanopore. Path 2 along the surface of the graphene has elevated K^+^ and decreased Cl^−^ concentration. (**d**) K^+^ (blue) and Cl^−^ (red) current densities plotted along the two illustrative paths shown in **b**. Path 2 shows much greater K^+^ current than Cl^−^ current, which results in K^+^/Cl^−^ selectivity. The overall pore selectivity results from these highly selective, highly conductive current paths. (**e**) Comparison of measured reversal potentials (black dots) and numerical predictions from the model with surface charge density of 0 C m^−2^ (blue line) and −0.6 C m^−2^ (red line). Both experimental data and numerical predictions are for a 10:100 mM concentration gradient. Right side, *y*-axis shows selectivity ratios calculated from the reversal potentials.

To test the plausibility of this hypothesis, we measured pore conductance with various KCl concentrations at pH 2 and 8 (Fig. 4a). These measurements were taken on an 8.5 nm diameter nanopore, the same pore for which TEM imaging was shown in Fig. 1b,c. The presence of charged, deprotonatable surface groups on the entire graphene surface would cause the conductance at pH 8 to be significantly higher than at pH 2, even at salt concentrations high enough that the surface charge is screened out (Debye length<pore radius). The conductance data shown in Fig. 4a clearly shows this effect, with conductance at pH 8 considerably higher than at pH 2 for all concentrations below 3 M.

To quantitatively test the surface charge hypothesis, we numerically solved the Poisson–Nernst–Planck (PNP) equations for a 2D axisymmetric pore geometry with a variable surface charge on the suspended graphene surface (see Supplementary Fig. 1 for details). The conductance data agrees with a model of an 8.5 nm pore with surface charge *σ*=−0.6 C m^−2^ (Fig. 4a). Initially, this surface charge density seems surprisingly high, considering that pristine graphene should not have any deprotonatable surface groups. However, it is possible that the high voltage pulses used to fabricate the nanopore oxidize the graphene surface, as well as hydrocarbon contaminants on the graphene surface^34^,^35^. As a comparison, graphene lightly treated with oxygen plasma was measured to have a surface charge density of *σ*=−0.24 C m^−2^ at pH 7 (ref. 36). To illustrate how this surface charge density causes high K^+^/Cl^−^ selectivity in this system, we examined two example paths in the numerical simulation (Fig. 4b): along the centre of the pore (path 1, Fig. 4b dotted line) and along the surface of the graphene (path 2, Fig. 4b, solid line). A plot of ion concentrations along the paths (Fig. 4c) shows increased K^+^ concentration and decreased Cl^−^ concentration along the entirety of the surface path (path 2). As a result, the K^+^ current density is elevated and the Cl^−^ current density reduced over the surface path (Fig. 4d). K^+^/Cl^−^ selectivity is therefore a result of both increased K^+^ current and reduced Cl^−^ current. Since these highly selective surface current paths are also highly conductive due to the elevated cation concentrations, they can cause even large pores to be very selective.

To quantitatively evaluate whether this model can account for the large measured selectivities, we simulated reversal potential measurements for a 10:100 mM concentration gradient and directly compared them with the experimental results shown in Fig. 2. Figure 4e shows the experimentally measured and numerically simulated reversal potentials as a function of pore size. Without the surface charge on the graphene membrane, the reversal potential and selectivity drop rapidly for pores larger than 1 nm, but the simulation including the surface charge models the measured data much better, predicting K^+^/Cl^−^ selectivity ratios above 100 for pores as large as 5 nm. The spread in experimental data most likely indicates that the surface charge and pore shape vary from sample-to-sample.

With this surface charge mechanism for ion selectivity in mind, we must be careful in the interpretation of reversal potential data using the GHK equation (equation 2). In the GHK model, the ion selectivity does not depend on salt concentration. However, the surface charge model implies that selectivity would decrease with increasing salt concentration, because the surface charge is screened out more strongly in high salt solutions (Supplementary Fig. 2). The reduction of selectivity at high salt concentrations, an effect called salting-out, has previously been observed and modelled in biological porins^37^. To determine if the surface charge model accurately predicts this effect, we measured the reversal potential for a 10:1 concentration ratio (*c*_high_/*c*_low_=10) as a function of salt concentration (*c*_high_) and compared the results with predictions from the PNP model (Supplementary Fig. 3). Indeed, the reversal potential (and therefore selectivity) is lower at higher salt concentrations, although there is significant sample-to-sample variability on how much the selectivity drops off at salt concentrations of 1 M or higher. This trend agrees with predictions from the numerical PNP model (black line), which includes charge screening effects. Therefore, while the GHK model is useful for estimating selectivities at a given salt concentration, it cannot be interpreted as a full physical model because it does not encapsulate the vital electrostatic effects of surface charge. In comparison, the numerical PNP model is a more complete model but does not offer the convenience of analytical solutions.

In summary, we have shown that graphene nanopores up to about 20 nm in diameter show K^+^/Cl^−^ selectivity ratios over 100 and monovalent/divalent cation selectivities up to 5. The K^+^/Cl^−^ selectivities can be explained by elevated concentrations of mobile cations near the graphene surface. Future work studying the source of these increased surface concentrations (which may include mechanisms other than the fixed surface charge examined here) is still needed to complete our understanding of the mechanism responsible for ion selectivity. Modifying the surface or using different thin materials may also allow nanopores to select for anions instead of cations. Although we have limited this study to single pores, we expect that large-area porous membranes containing many nanopores, 20 nm or smaller, will retain high selectivity while supporting orders of magnitude larger ionic currents. Previous investigations of the desalination potential of graphene have focused on subnanometer diameter pores for RO, but the results shown here suggest that such strict fabrication limitations are not necessary. The loosening of the pore size upper boundary from around 1 to 20 nm means that existing techniques for creating porous graphene membranes can likely be used to create highly effective cation exchange membranes for electrodialysis. Furthermore, these surprising observations indicate that atomically thin nanopores can behave quite differently than their thicker counterparts, and should continue to be a rich platform for studying nanoscale mechanisms of ion transport.

## Methods

### Graphene membrane preparation

Single-layer graphene was grown on Cu foil (Alpha Aesar) at 1,000 °C with a flow of 10 sccm H_2_ and 4 sccm CH_4_ for 40 min. Graphene was transferred to an approximately 150-nm diameter aperture in a 300-nm thick low-stress LPCVD silicon nitride (SiN_x_) membrane using established wet transfer techniques^15^. This SiN_*x*_ membrane was prepared using standard techniques, including photolithography and anisotropic KOH etching of the silicon substrate. The 150-nm diameter apertures were milled using an FIB (FEI/Micrion Vectra 980, 50 kV Ga^+^). Once transferred, samples were annealed at 250 °C for at least 2 h under 200 sccm H_2_ and 400 sccm Ar flow to remove surface contamination and were stored in a nitrogen-flushed drybox. Before wetting, the graphene membrane samples were annealed at 700 °C for 2 h under 200 sccm H_2_ and 400 sccm Ar to further remove surface contamination.

### Pore fabrication and fluidic cell measurements

Samples were loaded into an airtight PEEK fluidic cell with PDMS gaskets, and 99.999% pure CO_2_ was flowed through the flow-cell for 3 min to purge out any air. The fluidic cell was then flushed on both sides of the membrane with 1 M KCl, pH 10, which reacts with CO_2_ gas to form soluble carbonate anions, removing all gas in the fluidic cell and fully wetting the graphene membrane. The fluid reservoirs on each side of the fluidic cell were contacted via Ag/AgCl electrodes in 1 M KCl via agarose salt bridges to eliminate the potential generated from redox reactions on electrodes in asymmetric salt conditions. An Axopatch 200B patch clamp amplifier was used to apply d.c. voltage biases and measure ionic current. Pores were nucleated and enlarged using a pulse generator (HP8110A) as previously described^15^. Electrolyte solutions were prepared and buffered with 10 mM Tris, with the exception of 1 mM salt solutions, which were unbuffered. Pore diameters were estimated based on the conductance measurements using equation 2. It is worth noting that the additional conductance due to surface charge on the graphene surface is not taken into account in equation 2, so estimations of pore size based on the conductance measurements taken with neutral or basic pH solution may overestimate the pore size by as much as 50%.

### Ion selectivity measurements

Reversal potentials were determined from interpolated *I-V* measurements. Selectivity *S*_GHK_ was estimated by nonlinear least squares regression to the measured reversal potential using equation 1. Replicate measurements of reversal potential produced values with a standard deviation of about 3 mV. *S*_GHK_ depends exponentially on reversal potential and for the extremely high selectivities measured here (*S*_GHK_>100), small deviations in reversal potential produce large, nonlinear changes in *S*_GHK_. Error estimates in Fig. 2c,d were determined using Monte Carlo regression analysis on synthetic data sets, assuming a normal distribution of error in reversal potential measurements with a standard deviation of 3 mV. Reported error bars are the 5th and the 95th percentile of the resulting distribution of estimates of *S*_GHK_. Reported error bars in selectivity ratio *S*_i_ in Fig. 3c are the standard errors of the mean from the linear least-squares regression from two repeated measurements.

### TEM imaging

After electrical pulse fabrication and solution-based experiments, samples were removed from the fluidic cell and stored in deionized water. To avoid membrane damage due to surface tension, samples were critical-point dried before imaging. The nanopores were imaged in a JEOL 2010 F TEM operating at 200 kV. Efforts were made to minimize the beam exposure during imaging, because 200 kV electrons at high doses are capable of creating defects in graphene membranes. The yield for TEM imaging of electrical pulse-fabricated nanopores was very low (about 1 in 10) due to contamination of the membrane during drying and imaging. Contaminants from the solutions and from the air can easily cover the nanopores, especially under the electron beam, which can cause further deposition of contaminants. It seems that membranes that have been exposed to solution are more contamination-prone than membranes that have never been wet.

### Numerical modelling

Numerical solutions to the PNP equations were calculated using the COMSOL Multiphysics software (COMSOL, Inc.). The model solves for concentrations of K^+^ and Cl^−^ ions and the electric field using the steady-state Poisson equation and the Nernst–Planck equation for each ion individually (see Supplementary Fig. 1 and Supplementary Note 1). The negatively charged edge groups on the pore edge and the surface attraction to cations were modelled by imposing surface charge boundary conditions at the pore edge and on the graphene surface, respectively.

## Additional information

**How to cite this article:** Rollings, R. C. *et al.* Ion selectivity of graphene nanopores. *Nat. Commun.* 7:11408 doi: 10.1038/ncomms11408 (2016).

## Supplementary Material

Supplementary Information Supplementary Figures 1-3, Supplementary Table 1, Supplementary Note 1 and Supplementary References
