# Supplementary material for: Ion selectivity of graphene nanopores
Source: Nat Commun. 2016 Apr 22;7:11408. doi: 10.1038/ncomms11408 (PMC4844701; doi:10.1038/ncomms11408)
Supplement: Supplementary Information — Supplementary Figures 1-3, Supplementary Table 1, Supplementary Note 1 and Supplementary References [file ncomms11408-s1.pdf]

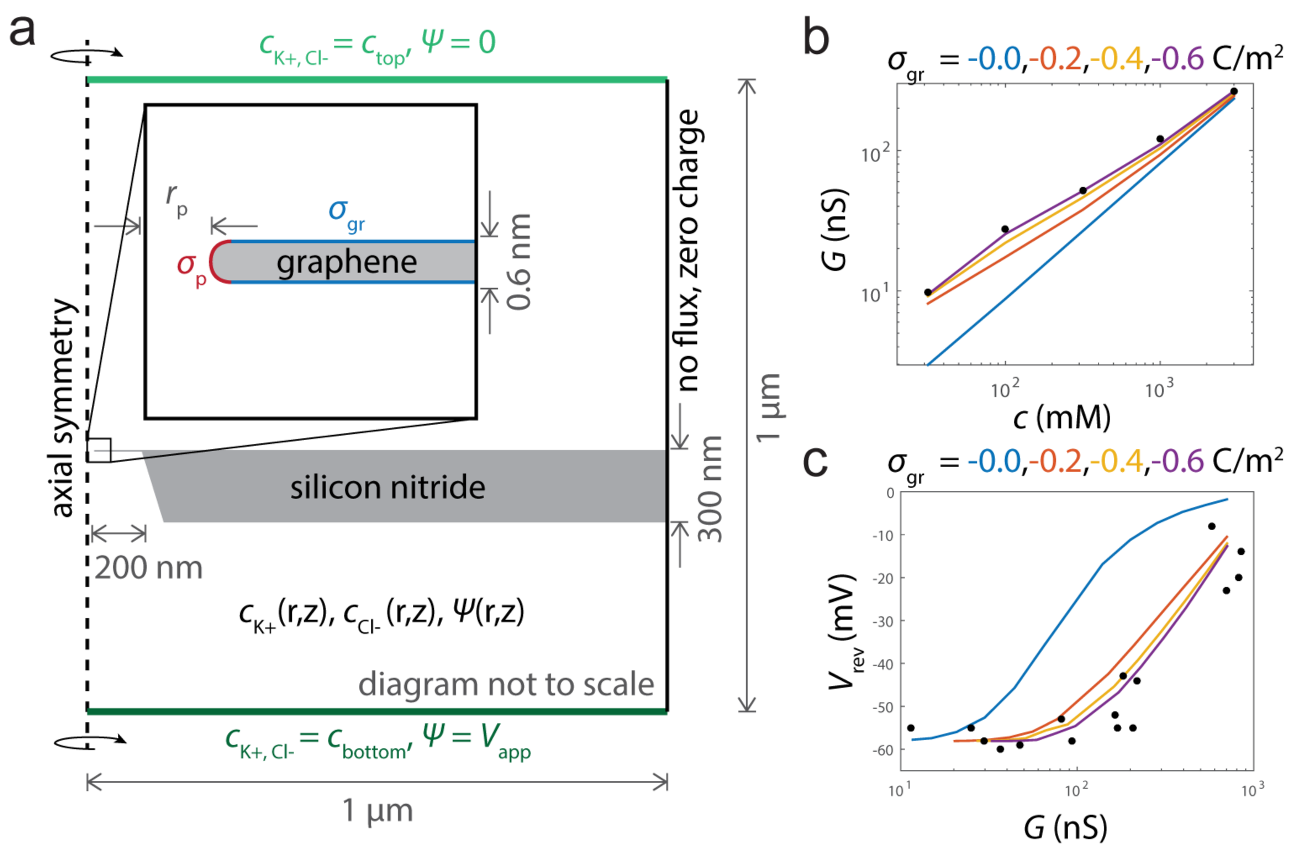

Supplementary Figure 1: Numerical PNP Model of Nanopore with Surface Charge.

Supplementary Figure 1a shows a diagram of the Poisson-Nernst-Planck system used to model the nanopore in the paper (Figure 4), solved using COMSOL Multiphysics Software. Supplementary Figure 1b shows model predictions for the conductance of an 8.5 nm nanopore compared with the experimental data conductance data taken at pH 8 (the data shown in Fig. 4a). Supplementary Figure 1c shows model predictions for the reversal potential in a 10:100 mM concentration gradient compared to experimental data for many pores (the data shown in Fig. 4e). Several values of the surface charge density are plotted to demonstrate the effect of the surface charge. At high values of surface charge density (< -0.4 C nm^{-2}), the predictions for conductance and reversal potential are not very sensitive to surface charge. The best fit value (σ_{gr} = -0.6 C m^{-2}) should therefore be interpreted more as an order-of-magnitude estimate than a precise measurement of surface charge. Details of the equations solved in the model are in Supplementary Note 1.

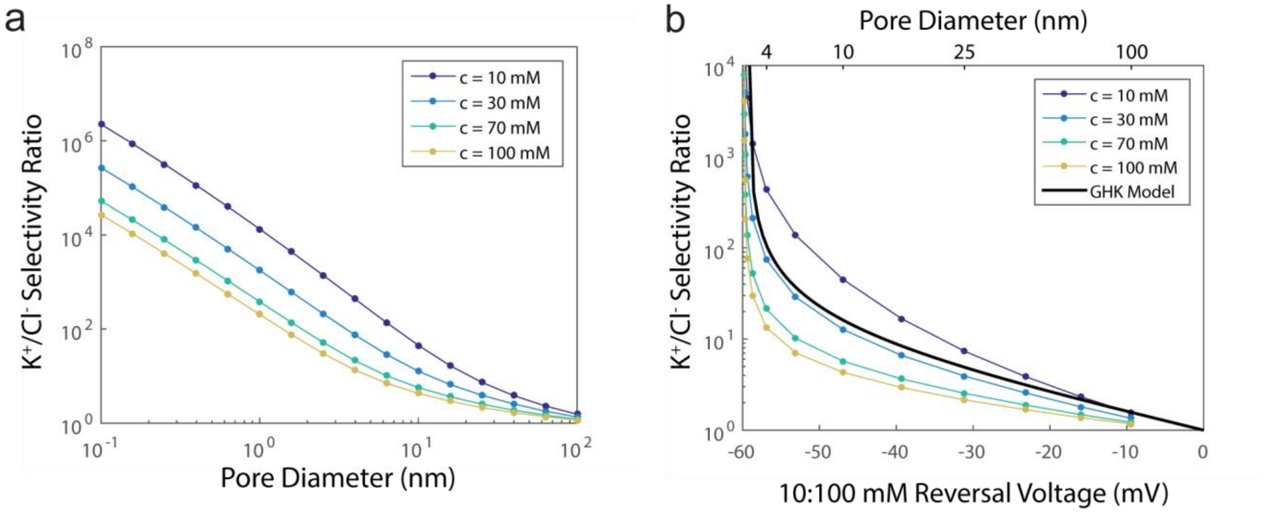

Supplementary Figure 2: Comparison of GHK and PNP Model Selectivity. Because the derivation of the GHK voltage equation contains several assumptions that may not apply to our graphene nanopore system[1], we must be careful in applying it to calculate selectivity. While the selectivity defined as the ratio of effective diffusion constants ($S_{\mathrm{GHK}} = D^*_{\mathrm{K+}}/D^*_{\mathrm{Cl-}}$) is well-defined in the context of the GHK model, this definition is not directly applicable to the more concretely-defined PNP system (see Supplemental Note 1), where the diffusion constants are given by bulk values (Supplementary Table 1). However, in the PNP model, we can directly define the selectivity as the ratio of K^+ current to Cl^- current $S_I = I_{\mathrm{K+}}/I_{\mathrm{Cl-}}$ measured in a symmetric salt situation ($c_{\mathrm{high}} = c_{\mathrm{low}}$). Unfortunately, this intuitive definition of selectivity cannot be used for the experimental data because K^+ and Cl^- currents cannot be independently measured. Here we test whether or not the two definitions of selectivity are equivalent. Supplementary Figure 2a shows numerical calculations of selectivity S_I as a function of pore size. Because counter-ion screening of the surface charge depends on the solution concentration, selectivity S_I is also dependent on solution concentration. The selectivities for lower

concentrations (30 and 10 mM) show selectivities around 100 persisting for pores as large as 5 nm, which agrees with the reversal potential calculations in Figure 4. In order to directly compare the two definitions of selectivity, we plotted S_{GHK} and S_I as a function of reversal potential in Supplementary Figure 2b. The black line shows S_{GHK} calculated using the GHK voltage equation with reversal potentials that were calculated from the PNP model with a 10 mM:100 mM concentration gradient. The colored lines show S_I calculated directly from the same PNP model without a concentration gradient. $S_I(c)$ is concentration dependent, whereas S_{GHK} is not. However, for $c = 30$ mM (approximately the geometric mean of $c_{\mathrm{high}}$and c_{low}), S_I is very similar to S_{GHK}. Therefore, we conclude that the GHK voltage equation is an acceptable model for calculating K^+/Cl^- selectivity, with the understanding that $S_{\mathrm{GHK}} \cong S_{\mathrm{PNP}}(\sqrt{c_{\mathrm{high}} c_{\mathrm{low}}})$.

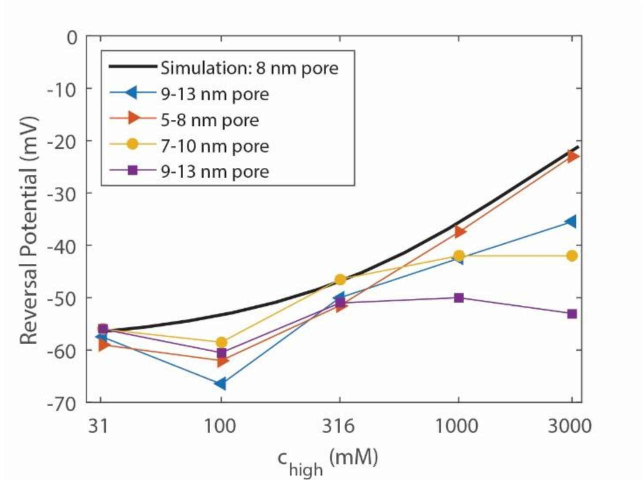

Supplementary Figure 3: Salting Out of Ion Selectivity. In the GHK model, the reversal potential depends on the bulk concentration ratio ($c_{\mathrm{high}}/c_{\mathrm{low}}$) but not on the absolute value of the concentrations (c_{high}). In other words, selectivity does not depend on salt concentration. However, the surface charge model implies that selectivity decreases with increasing salt concentration because the surface charge is screened out more strongly in high salt solutions. The reduction of selectivity at high salt concentrations, an effect called "salting-out", has previously been observed and modelled in biological porins[2]. To determine if the surface charge model accurately predicts this effect, we measured the reversal potential as a function of salt concentration for several nanopores with a 10:1 concentration ratio and compared the results to predictions from the PNP model (Supplementary Figure 1). Indeed, the reversal potential (and therefore selectivity) is lower at higher salts, although there is significant sample-to-sample variability on how much the selectivity drops off at salt concentrations of 1M or higher. This trend agrees with predictions from the numerical PNP model (black line), which includes charge screening effects.

Ion	**Electrophoretic Mobility** μ_i [10^{-4} (cm s^{-1}) / (V cm^{-1})]	**Relative Mobility** μ_i / μ_{K^+}
K^+	7.62	1.00
Cl^-	7.92	1.04
Li^+	4.01	0.53
Na^+	5.19	0.68
Cs^+	8.01	1.05
Ca^{2+}	6.17	0.81
Mg^{2+}	5.50	0.72

Supplementary Table 1: Ion Mobilities. Electrophoretic mobilities for ions studied in the paper. These values are from Hille et al.[1] The mobility relative to K^+ has also been tabulated to quantify the relative variation of mobilities amongst the different ions.

Supplementary Note 1. Numerical Modeling of Poisson-Nernst-Planck Equation

The model as shown in Supplementary Figure 1 solves for three scalar fields, the concentrations c_{K+} and c_{Cl-}, and the electric potential ψ, given Poisson's equation and the steady-state Nernst-Planck Equation for each ion.

$$\nabla^2\psi = \frac{eF}{\epsilon}(c_{\mathrm{K}^+} - c_{\mathrm{Cl}^-}) \tag{1}$$

$$\nabla \cdot J_{\mathrm{K}^+} = \nabla \cdot \left[-FD_{\mathrm{K}+}\nabla c_{\mathrm{K}^+} - \frac{F^2 D_{\mathrm{K}+}}{RT} c_{\mathrm{K}^+}\nabla\psi \right] = 0 \tag{2}$$

$$\nabla \cdot J_{\mathrm{Cl}^-} = \nabla \cdot \left[FD_{\mathrm{Cl}-}\nabla c_{\mathrm{Cl}^-} - \frac{F^2 D_{\mathrm{Cl}-}}{RT} c_{\mathrm{Cl}^-}\nabla\psi \right] = 0 \tag{3}$$

where e is the electron charge, F is Faraday's constant, ϵ is the relative permeability, J_i is the current density for the ion i, D_i is the diffusion constant for the ion i, R is the universal gas constant, and T is temperature. The relevant physical dimensions and boundary conditions are indicated on the diagram. Surface charge boundary conditions were imposed on the pore edge (σ_{p} = -1 C m^{-2}) and on the graphene surface (σ_{gr}, variable) to model deprotonatable chemical groups on the pore edge and graphene surface, respectively.

Supplementary References

1. Hille, B. *Ion Channels of Excitable Membranes*, *Third Edition*. (Sinauer Associates, 2001).

2. Alcaraz, A., Nestorovich, E. M., Aguilella-Arzo, M., Aguilella, V. M. & Bezrukov, S. M. Salting out the ionic selectivity of a wide channel: the asymmetry of OmpF. *Biophys. J*. **87,** 943–957 (2004).
